# Predicting future uncertainty constraints on global warming projections

**DOI:** 10.1038/srep18903

**Published:** 2016-01-11

**Authors:** H. Shiogama, D. Stone, S. Emori, K. Takahashi, S. Mori, A. Maeda, Y. Ishizaki, M. R. Allen

**Affiliations:** 1 Center for Global Environmental Research, National Institute for Environmental Studies, 16-2 Onogawa, Tsukuba, Ibaraki 305-8506, Japan; 2 Computational Research Division, Lawrence Berkeley National Laboratory, Berkeley, CA 94720, USA; 3 Center for Social and Environmental Systems Research, National Institute for Environmental Studies, 16-2 Onogawa, Tsukuba, Ibaraki 305-8506, Japan; 4 Department of Industrial Administration, Faculty of Science and Technology, Tokyo University of Science, Chiba 278-8510, Japan; 5 Graduate School of Arts and Sciences, The University of Tokyo, 3-8-1 Komaba, Meguro-ku, Tokyo, 153-8902, Japan; 6 School of Geography and the Environment, University of Oxford, OX1 3QY, Oxford, UK; 7 Department of Physics, University of Oxford, OX1 3QY, Oxford, UK

## Abstract

Projections of global mean temperature changes (Δ*T*) in the future are associated with intrinsic uncertainties. Much climate policy discourse has been guided by “current knowledge” of the Δ*T*s uncertainty, ignoring the likely future reductions of the uncertainty, because a mechanism for predicting these reductions is lacking. By using simulations of Global Climate Models from the Coupled Model Intercomparison Project Phase 5 ensemble as pseudo past and future observations, we estimate how fast and in what way the uncertainties of Δ*T* can decline when the current observation network of surface air temperature is maintained. At least in the world of pseudo observations under the Representative Concentration Pathways (RCPs), we can drastically reduce more than 50% of the Δ*T*s uncertainty in the 2040 s by 2029, and more than 60% of the Δ*T*s uncertainty in the 2090 s by 2049. Under the highest forcing scenario of RCPs, we can predict the true timing of passing the 2 °C (3 °C) warming threshold 20 (30) years in advance with errors less than 10 years. These results demonstrate potential for sequential decision-making strategies to take advantage of future progress in understanding of anthropogenic climate change.

The large uncertainty associated with projections of future climate change is one of the barriers to political agreement on mitigation policy. Fig. 1a shows the 10–90% uncertainty ranges of global mean temperature changes (Δ*T*s; relative to the 1900–1919 mean) based on a Gaussian fit to 15 global climate models (GCMs) (Supplementary Table 1)that contributed to the Coupled Model Intercomparison Project Phase 5 (CMIP5)^1^. A promising approach to obtain a confidence range of a Δ*T* is the Allen, Stott, and Kettleborough (ASK) method^2^,^3^,^4^,^5^. The basic idea of the ASK method is simple: if a GCM overestimates the observed magnitude of historical climate change (not only the global mean change, but also spatiotemporal patterns), such a GCM will overestimate future climate changes by a proportional amount, and vice versa (see Methods). By considering the uncertainties arising from the internal natural variability of the climate system, one can obtain observationally constrained confidence ranges of future ∆*T*s. Various approaches including the ASK method have provided the “current knowledge” of future projections that have guided mitigation and adaptation studies^1^,^6^,^7^.

The uncertainty ranges of ∆*T*s are expected to decline in the future thanks to new observations, greater warming signals, and further progress in understanding of the climate system. Although prediction of future advancements of scientific knowledge is considered to be difficult, it is possible to estimate the importance of future observational monitoring. Here we will demonstrate how fast and in what way the uncertainties of ∆*T*s can decline as a result of future updates of surface air temperature (SAT) observations when the current observation network of SAT is maintained. Stott and Kettleborough^3^ considered the ∆*T*s of a single GCM for the period 2000–2019 as updated pseudo observations (POs) (combined with the real observations prior to 2000) and applied the ASK method to demonstrate the possible reduction of the uncertainty ranges of the ∆*T*s. We extend this idea of analysis by using POs derived from each simulation in the multi-GCM ensemble (see Methods). While some previous studies using simple climate models have estimated learning rates by analyzing future POs from their simple models, their results are sensitive to the assumption of natural variability, which can not be simulated by simple models, and prior distributions of climate parameters^8^,^9^,^10^. These limitations of simple model analyses cause difficulties in usage of their estimates of learning rates in mitigation studies. Our study is the first to comprehensively address the rates of future learning using an ensemble of full-GCMs. It has been suggested that learning process using limited observations may result in a sizable discrepancy between the observationally constrained projections and the truth, i.e., the uncertainty bounds of projection can converge to a wrong answer^10^. We first investigate lead-time within that the observationally constraints work well.

The previous studies of sequential decision-making strategies have generally used hypothetical strong assumptions about uncertainties in climate change projections, e.g., the perfect knowledge of climate sensitivity in 2030^11^,^12^,^13^,^14^. We provide the information of the more plausible future reduction of uncertainties in climate change projection that can be useful for studies of sequential decision-making strategies.

## Results

**Figure 1:**
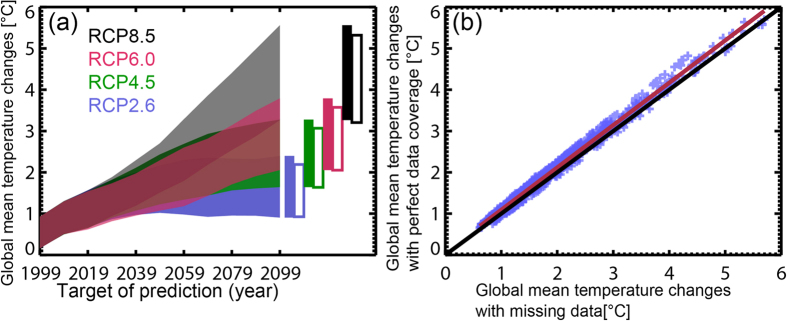
Raw GCM projections of global mean temperature change. (**a**) Decadal mean projections of Δ*T* with perfect data coverage are shown for each RCP for the 10–90% range of the Gaussian distributions of GCMs (°C; relative to the 1900–1919 mean). The filled and unfilled vertical bars show the corresponding 10–90% ranges of Δ*T* in the 2090 s with perfect data coverage and with past and sustained current coverage, respectively. (**b**) Blue crosses indicate decadal mean Δ*T* (°C) during the 1990 s–2090 s for all GCMs and RCPs with perfect data coverage (vertical axis) and with past and sustained current coverage (the horizontal axis). The black line is the one-to-one line, and the red line is the regression line.

Lack of SAT observations in the polar regions leads to underestimations of global warming^15^ and larger uncertainty (Fig. 1a). The original ASK method does not take into account the biases caused by gaps in the spatial coverage of SAT monitoring. Here we adjust the ASK predictions by using the regression relationship between “∆*T*s with perfect data coverage” and “∆*T*s with missing data” (Fig. 1b) (see Methods). Supplementary Fig. 1a is an example of changes in the 10–90% uncertainty bounds of ∆*T*s, which are estimated by applying the ASK method with updated POs. The projected ∆*T*s of the POs all the way through to the 2090 s are within the uncertainty bounds of ASK predictions based on POs prior to 2009. As the observation record is updated, the uncertainty range of the ∆*T*s decreases, as expected.

In contrast, in the case of Supplementary Fig. 1b, the 10–90% error bounds of the ASK predictions based on POs prior to 2009 or 2029 include the future ∆*T*s of the POs with a lead time of a few decades, but underestimated the ∆*T*s in the latter half of the 21st century. These downward biases of the ASK predictions are caused by the fact that the ∆*T* of the multi-model average (MMA) is smaller than the ∆*T* of the PO until 2029 and larger after that. When the observations are updated to 2049, the errors of the ASK predictions are corrected, and the predicted ∆*T* for 2099 falls within the uncertainty bound. These results suggest that observations prior to 2009 or 2029 are not enough to adequately constrain ∆*T*s for the latter half of this century. One possible reason is that the relative contributions of greenhouse gases and other forcing factors (e.g., anthropogenic aerosols) are different between the past and the latter half of this century (discussed later).

**Figure 2:**
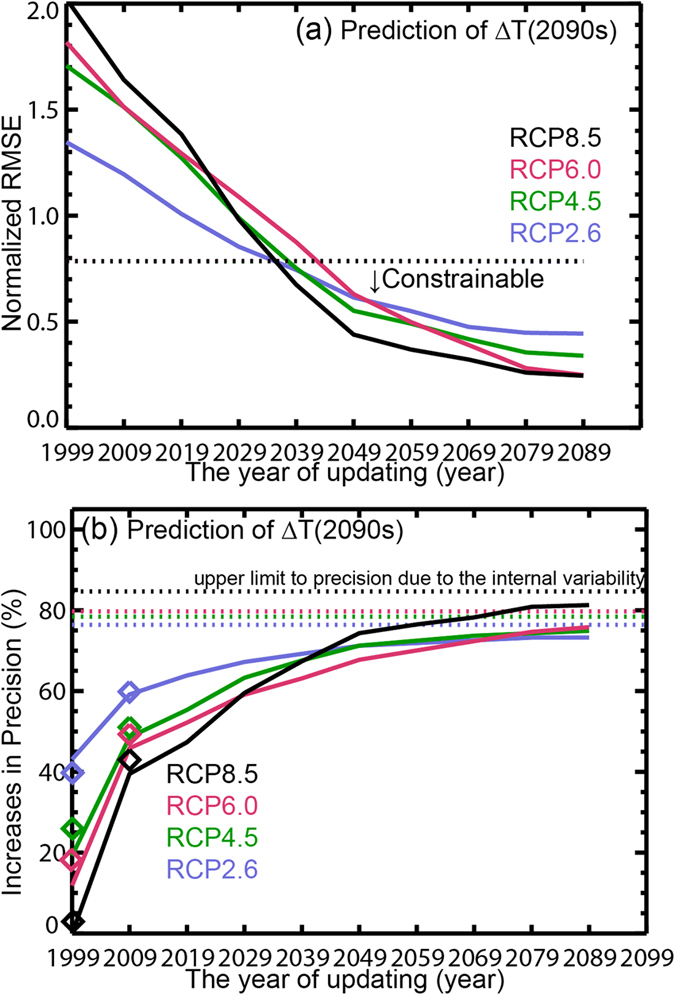
Normalized root mean square errors and increases in precision of Δ*T*s in the 2090 s. (**a**) Normalized root mean square errors for the year of updating (the horizontal axis) and for each RCP. The black dotted line indicates the threshold of the constrainable level. (**b**) Increases in precision (%) for the year of updating (the horizontal axis) and for each RCP. Diamonds show the increases in precision estimated by using the real observations for the year 1900–1999 and 1910–2009 periods (see Methods). The dotted lines show the upper limits of precision determined by the internal climate variability.

The ASK method is applied for all possible POs chosen from each of the CMIP5 climate model simulations for each RCP. The results of Supplementary Fig. 1 demonstrate that we should investigate not only the estimated rate of decreases of the uncertainty ranges, but also whether the ASK predictions accurately constrain the ∆*T*s or lead to additional systematic errors in the ∆*T*s predictions. We compute the root mean square errors (RMSEs) for the 50%-tile prediction for the raw ensemble (RMSE0) and the ASK predictions (RMSE1) (Methods). Normalized RMSE (NRMSE) is defined as RMSE1/RMSE0. If NRMSE is larger than 1, the ASK method caused systematic biases. If NRMSE is smaller than 1 with statistical significance at the 10% level (estimated using an *F*-test), we consider the future ∆*T*s are observationally “constrainable” (i.e., we can reduce errors involving systematic biases relative to the truth at the given statistical significance level). Previous learning studies have not investigated well whether the future ∆*T*s are constrainable or not^10^. Figure 2a indicates the NRMSEs for the ∆*T*s in the 2090s as functions of the year of updating. An important finding is that observations of SAT up to 2009 or 2019 are not enough to satisfactorily predict ∆*T*s in the 2090 s, rather it is necessary to wait until 2049 to accurately reduce errors.

Once the ASK predictions become constrainable, this method has great power to decrease the spreads of ∆*T*s. We refer to “increases in precision (IPs)” as the fractional decreases of the 10–90% uncertainty range in the ASK predictions relative to that in the raw projection (Fig. 2b). Observations up to 2049 result in IPs of more than 60% for the ∆*T*s in the 2090 s, respectively. The IPs approach asymptotically the upper limits determined by the climate internal variability. To exceed these limits, we need forecast techniques that initialize the state of the ocean^16^, which are not the focus of this study. Gillet *et al.*^4^ claimed that a detection and attribution method using past real observations up to 2010 can reduce the half range of transient climate response (∆T responses to a doubling CO_2_ concentration) of the CMIP5 ensemble. It is consistent with our results, i.e., the IPs using the POs prior to 2009 are roughly 40%–60% and close to the IPs based on the real observations (diamonds in Fig. 2b). However the previous studies have not well investigated the constrainable lead time. In a later section, we will discuss a possibility to extend the constrainable lead time using the past and near future observations.

**Figure 3:**
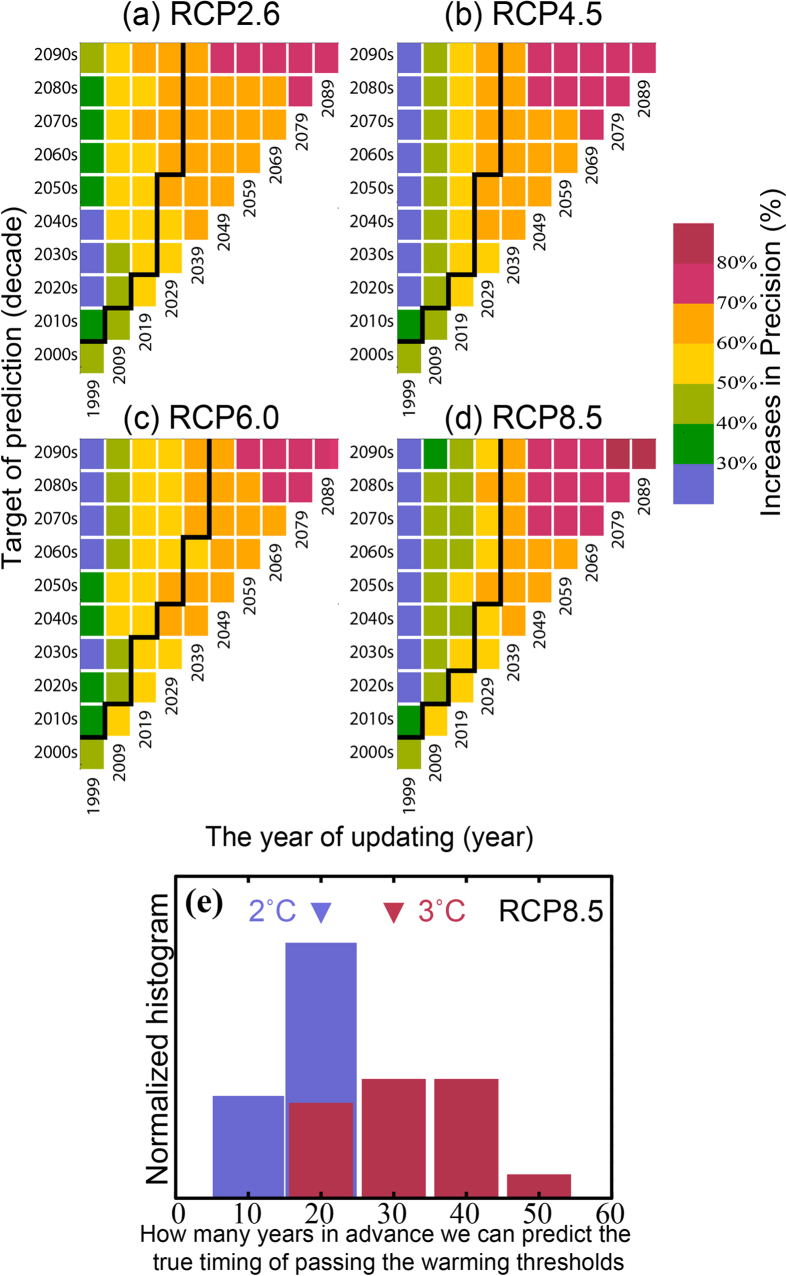
Summary of constrainable lead times and increases in precision. Color shading indicates increases in precision (%) for each year of updating (horizontal axis) and target of prediction (vertical axis) from (**a**) RCP2.6 to (**d**) RCP8.5. Predictions below and right of the black lines are constrainable according to our threshold for accurate prediction. (**e**) How many years in advance we can predict the true timing of exceeding the 2 °C and 3 °C warming thresholds. Blue and red bars show the normalized histograms for the 2 °C and 3 °C warming thresholds under RCP8.5, respectively. Triangles indicate the median values.

Figures 3a–d summarize the constrainable lead time and IPs as functions of the updating year and the target period of prediction. The constrainable lead times are 10–20 years until 2019. As more observations are accumulated, the constrainable lead times tend to be longer. The Δ*T*s of the 2040 s can be constrainable by 2029, and the corresponding IPs are about 50–70%. By combining our Δ*T*s predictions and pattern scaling methods^17^, we can provide likelihood distributions of regional climate predictions applicable to impact and adaptation studies. In other words, for studies of adaptation strategies until the middle of this century, we can take advantage of Δ*T* predictions with lead times of 20 years, which have less than half the uncertainty of raw GCM spreads. Updating SAT observations up to 2049 allow us to constrain about 60–80% of the uncertainty in the Δ*T* for the 2090 s.

The POs for RCP8.5 pass the 2 °C and 3 °C thresholds during 2030 s–2060 s and 2050 s–2090 s, respectively (Fig. 1). Additional observations would enable us to correctly predict the true timing of exceeding the 2 °C threshold with errors less than one decade 20 years in advance (the median; the min-max range is 10–20 years), and the 3 °C threshold 30 years (20–50 years) in advance (see Method) (Fig. 3e).

## Discussion

Some studies have tried to incorporate the effects of possible future learning about climate change into mitigation analyses^8^,^11^,^12^,^13^,^14^. Most of such studies have used idealized, optimal assumptions of learning speed, an example being that there is perfect knowledge of climate sensitivity in the 2030 s^12^. The analysis applied here would allow the investigation of sequential decision-making strategies based on an estimate of future learning more plausible than the hypothetical perfect knowledge assumption^13^. Of course, such a future learning scenario does not suggest inaction on mitigation efforts until further information is obtained. Global negotiation efforts are currently focused on keeping future global mean temperatures to no more than 2 °C above pre-industrial levels. The latest carbon dioxide emissions are higher than the RCP8.5 scenario^18^. Further paralysis of global mitigation efforts would rapidly close the feasibility window to keep ∆*T* below the 2 °C target^19^,^20^.

In this paper we have substituted a standard assumption that simulations of current climate models span the range of possible future climate change^1^. While this assumption lacks a direct observable reference^21^, it nevertheless relies on some capability of the current generation of climate models. Beyond this bias, however, we have only considered the increases in the potential of observational constraints gained through continuation of existing monitoring efforts, and not the application of knowledge gained from expanded monitoring, developments in climate modelling, or greater capacity to understand climate systems. Combining our method with different approaches and other observations^22^,^23^,^24^ would provide more information for learning. The likely gain of such knowledge would imply that accurate prediction might be obtainable at earlier dates than found in this study.

The constrainable periods abruptly increase when the observations accumulate to 2039 and later. It may be partly caused by that share of anthropogenic aerosols to the total radiative forcing rapidly decline during the first half of the 21st century in the RCPs^25^. Involving the POs during 2030 s and later, where the greenhouse gases dominate the total radiative forcing like that in 2090 s, may reduce the errors of the ASK predictions. The full ASK method, which scales the response of individual forcing agents, may increase the constrainable periods using the observations prior to 2029. However, such individual forcing simulations are not generally available from the current generation of climate models^5^. The individual forcing simulations are planned to be performed for Detection and Attribution Model Intercomparison Project^26^. Thus there is a possibility that these extra simulations enable us to largely reduce the uncertainty of ∆T projections in the next few years.

## Methods

We analyse the SAT simulations from 15 GCMs that have been used for historical simulations (1900–2005) and all four RCP simulations (2006–2099) of the CMIP5 database (Supplementary Table 1). We also use preindustrial control runs (CTL) of the 15 GCMs (400 years for each GCM). The horizontal resolution of GCM data is interpolated to 5° × 5°. When and where values are missing in the HadCRUT4^27^ data, the GCM data are also set to missing for the ASK analyses. During 2010–2099, the observation mask of the GCM data is set at 2009. Ten-year averaged values (1900 s, . . , 2090 s) for each grid are computed if 6-month, 5-year, or 6-decade data are available for each year, each decade, and the 1900–2009 period, respectively; otherwise, such a grid point is filled with a missing value.

The ASK method is based on a formal detection and attribution analysis technique^28^,^29^. Here *Y* and *X* denote the spatiotemporal patterns of the PO data and the MMA data. *Y* and *X* are anomalies from the averages during the training periods (the training periods are explained later). To improve signal-to-noise ratios, we apply a >5000 km spatial scale smoothing for *Y* and *X*. *Y* and *X* are projected onto the leading orthogonal modes of internal climate variability, which have been deduced from 15 × 200-year CTL simulations^30^. We applied a total least squares regression version^29^:


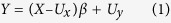


where β is the scaling factor, and *U*_*y*_ and *U*_*x*_ are the internal climate variability components of *Y* and *X*, respectively. We estimate uncertainties in β due to *U*_*y*_ and *U*_*x*_ using the other 15 × 200-year CTL simulations. The scaling factor β gives a measure of how well *X* simulates the magnitude of climate change signals in Y: if β is <1, *X* overestimates the signal; if β is >1, *X* underestimates the signal. We resample GCMs 100 times with replacement to compute multi-model averaged *X* values to take account of inter-GCM diversity.

We test whether the variance of the residual of equation (1), [*Y–(X–U*_*x*_)β], is consistent with that of *U*_*y*_^28^. We use maximum truncation numbers (<15) of leading orthogonal modes that pass the residual variance tests (*F*-test at *p* = 0.1)^28^. If the residual variance tests fail, or β is not well constrained (β < 0 or β > 7) for all possible truncation numbers and initial condition ensemble members, we omit that GCM as a PO from the following analyses.

Use of PO data far from the target year (e.g., data from the 1900 s for predictions up to the 2090 s) results in smaller signal-to-noise ratios and different contributions of greenhouse gases and aerosols than the PO data close to the target year. The training period lengths of equation (1) are therefore restricted. We test the training period lengths from 50 to 100 years. For example, the 100-year training periods are 1900–1999, 1910–2009, .., and 1990–2089. Supplementary Fig. 1 shows the results for the 80-year training period. Because the fractions of failure in residual tests for all possible truncation numbers and initial condition ensemble members are less than 5% for 70–100 year training period lengths (Supplementary Fig. 2), we use 70-, 80-, 90- and 100-year training period lengths.

Having inferred the distribution of β from equation (1), we determine the likelihood distributions of future projections (*Y*^*ASK*^) by applying the ASK method^2^,^3^:


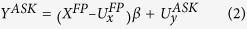


where *X*^*FP*^ are the future projections of the MMA (anomalies from the training period mean of Eq. 1). 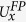 and 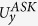 are the internal climate variabilities in *X*^*FP*^ and *Y*^*ASK*^, respectively, which are randomly produced (500 samples for each) by using Gaussian distributions with standard deviations (after taking account of the effects of averaging on the standard deviations) deduced from the CTL runs. We add the difference of POs between the training period average and the 1900–1919 mean to *Y*^*ASK*^. The full ASK method scales the response of individual forcings (well-mixed greenhouse gases and the other anthropogenic factors (e.g., anthropogenic aerosols)). However we use the response to the full forcing due to the lack of data availability of the individual forcing experiments.

We correct biases due to missing data by computing


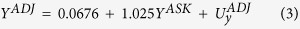


where the 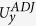
are 500-member random samples from a Gaussian distribution with the standard deviation of the regression residual (0.0834). *Y*^*ADJ*^ is computed for all the initial condition ensemble members of the PO GCM and combined in a single frequency distribution.

We investigate errors of the 50%-tile ASK prediction for each PO and each training length: 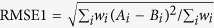 , where 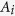 and 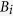 are the 50%-tile ASK prediction and the PO for the *i*-th sample of all the PO-GCMs × training-length combinations. 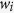 is the inverse of initial condition ensemble size. We also compute the root mean square errors of the raw ensemble mean prediction: 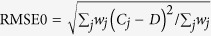, where 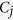 is the *j*-th PO, and *D* is the ensemble average. 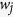 is the inverse of initial condition ensemble size. We define the normalized root mean square error (NRMSE) as RMSE1/RMSE0. We consider that the degree of freedom in the *F*-test of NRMSE is (15 × 4*–*1, 15*–*1).

The increases in precision (IPs) shown in Figs 2 and 3a–d are the averages of IPs for all the PO models and training period lengths. There are not large variations of IPs across the PO models and the training period lengths (Supplementary Fig. 3). We also show the IPs estimated by analyzing the 100 realizations of HadCRUT4^27^ for the year 1900–1999 and 1910–2009 periods in Fig. 2b and Supplementary Fig. 3.

We examine the year of updating when both the 10% and 90% bound values of exceedance time of the 2 °C or 3 °C threshold for the ASK prediction first fall within ±1 decade of the true timing after that the ∆*T* in the true timing become constrainable. We compute the averages across all the training period lengths. Figure 3e shows this as the number of years before the crossing date until an accurate prediction can be made.

## Additional Information

**How to cite this article**: Shiogama, H. *et al.* Predicting future uncertainty constraints on global warming projections. *Sci. Rep.* **6**, 18903; doi: 10.1038/srep18903 (2016).

## Supplementary Material

Supplementary Information

## References

[b1] Collins M. *et al.* Long-term Climate Change: Projections, Commitments and Irreversibility. In: Climate Change 2013: The Physical Science Basis. Contribution of Working Group I to the Fifth Assessment Report of the Intergovernmental Panel on Climate Change [ Stocker T. F., Qin D., Plattner G.-K., Tignor M., Allen S. K., Boschung J., Nauels A., Xia Y., Bex V. & Midgley P. M. (eds.)]. Cambridge University Press, Cambridge, United Kingdom and New York, NY, USA (2013).

[b2] Allen M. R., Stott P. A., Mitchell J. F. B., Schnur R. & Delworth T. L. Quantifying the uncertainty in forecasts of anthropogenic climate change. Nature 407, 617–620, doi: 10.1038/35036559 (2000). 11034207

[b3] Stott P. A. & Kettleborough J. A. Origins and estimates of uncertainty in predictions of twenty-first century temperature rise. Nature 416, 723–726, doi: 10.1038/416723a (2002). 11961551

[b4] Gillett N. P. *et al.* Improved constraints on 21st-century warming derived using 160 years of temperature observations, Geophys. Res. Lett. 39, L01704 (2012).

[b5] Stott P., Good P., Jones G., Gillett N. & Hawkins E. The upper end of climate model temperature projections is inconsistent with past warming. Environmental Research Letters 8, doi: 10.1088/1748-9326/8/1/014024 (2013).

[b6] IPCC: Summary for policymakers. In: Climate Change 2014: Impacts, Adaptation, and Vulnerability. Part A: Global and Sectoral Aspects. Contribution of Working Group II to the Fifth Assessment Report of the Intergovernmental Panel on Climate Change [ Field C. B., Barros V. R., Dokken D. J., Mach K. J., Mastrandrea M. D., Bilir T. E., Chatterjee M., Ebi K. L., Estrada Y. O., Genova R. C., Girma B., Kissel E. S., Levy A. N., MacCracken S., Mastrandrea P. R., & White L. L. (eds.)]. Cambridge University Press, Cambridge, United Kingdom and New York, NY, USA, pp. 1–32. (2014).

[b7] IPCC: Summary for Policymakers, In: Climate Change 2014, Mitigation of Climate Change. Contribution of Working Group III to the Fifth Assessment Report of the Intergovernmental Panel on Climate Change [ Edenhofer O., Pichs-Madruga R., Sokona Y., Farahani E., Kadner S., Seyboth K., Adler A., Baum I., Brunner S., Eickemeier P., Kriemann B., Savolainen J., Schlömer S., von Stechow C., Zwickel T. & Minx J. C. (eds.)]. Cambridge University Press, Cambridge, United Kingdom and New York, NY, USA (2014).

[b8] Webster M., Jakobovits L. & Norton J. Learning about climate change and implications for near-term policy. Climatic Change 89, 67–85, doi: 10.1007/s10584-008-9406-0 (2008).

[b9] Urban N. M., Holden P. B., Edwards N. R., Sriver R. L. & Keller K. Historical and future learning about climate sensitivity. Geophysical Research Letters 41, 2543–2552, doi: 10.1002/2014gl059484 (2014).

[b10] Olson R. *et al.* What is the effect of unresolved internal climate variability on climate sensitivity estimates? J. Geophys. Res.-Atmos. 118, 4348–4358, doi: 10.1002/jgrd.50390 (2013).

[b11] Manne A. S. & Richels R. G. Buying Greenhouse Insurance, The MIT Press (1992).

[b12] Yohe G., Andronova N. & Schlesinger M. Climate - To hedge or not against an uncertain climate. Science 306, 416–417, doi: 10.1126/science.1101170 (2004). 15486278

[b13] Mori S., Matuo T. & Ohkura M. Minimum Regret Climate Policy with Act-Then-Learn Decision—A new model framework under long-term uncertainties. Journal of Energy and Power Engineering, 7 (2013), 1106 1115.

[b14] Neubersch D., Held H. & Otto A. Operationalizing climate targets under learning: An application of cost-risk analysis. Climatic Change 126, 305–318 (2014).

[b15] Cowtan K. & Way R. G. Coverage bias in the HadCRUT4 temperature series and its impact on recent temperature trends. Q. J. R. Meteorol. Soc. (2014).

[b16] Hawkins E. & Sutton R. The Potential to narrow uncertainty in regional climate predictions. Bull. Amer. Meteor. Soc. 90, 1095–1107 (2009).

[b17] Shiogama H. *et al.* Emission scenario dependencies in climate change assessments of the hydrological cycle. Climatic Change 99, 321–329 (2010).

[b18] Peters G. P. *et al.* The challenge to keep global warming below 2 degrees C. Nature Climate Change 3, 4–6 (2013).

[b19] Rogelj J., McCollum D. L., O’Neill B. C. & Riahi K. 2020 emissions levels required to limit warming to below 2 degrees C. Nature Climate Change 3, 405–412, doi: 10.1038/nclimate1758 (2013).

[b20] Allen M. R. & Stocker T. F. Impact of delay in reducing carbon dioxide emissions. Nature Climate Change 4, 23–26, doi: 10.1038/nclimate2077 (2014).

[b21] Knutti R. The end of model democracy? Climatic Change 102, 395–404, doi: 10.1007/s10584-010-9800-2 (2010).

[b22] Forest C. E., Stone P. H. & Sokolov A. P. Constraining climate model parameters from observed 20th century changes. Tellus Ser. A-Dyn. Meteorol. Oceanol. 60, 911–920, doi: 10.1111/j.1600-0870.2008.00346.x (2008).

[b23] Shiogama H. *et al.* Observational constraints indicate risk of drying in the Amazon basin. Nat. Communications 2, 253, doi: 10.1038/ncomms1252 (2011). 21448152

[b24] Sherwood S. C., Bony S. & Dufresne J.-L. Spread in model climate sensitivity traced to atmospheric convective mixing. Nature 505, 37–42, doi: 10.1038/nature12829 (2014). 24380952

[b25] Myhre G. *et al.* Declining uncertainty in transient climate response as CO2 forcing dominates future climate change. Nature Geoscience 8, 181–185 (2015).

[b26] Gillett N. & Shiogama H. Detection and Attribution MIP (DAMIP), http://www.wcrp-climate.org/modelling-wgcm-mip-catalogue/modelling-wgcm-mips/475-modelling-wgcm-damip (2014) (Date of access:03/09/2015).

[b27] Morice C. P., Kennedy J. J., Rayner N. A. & Jones P. D. Quantifying uncertainties in global and regional temperature change using an ensemble of observational estimates: The HadCRUT4 data set. J. Geophys. Res.-Atmos 117, doi: 10.1029/2011jd017187 (2012).

[b28] Allen M. R. & Tett S. F. B. Checking for model consistency in optimal fingerprinting. Climate Dynamics 15, 419–434, doi: 10.1007/s003820050291 (1999).

[b29] Allen M. R. & Stott P. A. Estimating signal amplitudes in optimal fingerprinting, part I: theory. Climate Dynamics 21, 477–491, doi: 10.1007/s00382-003-0313-9 (2003).

[b30] Jones G. S., Stott P. A. & Christidis N. Attribution of observed historical near-surface temperature variations to anthropogenic and natural causes using CMIP5 simulations. J. Geophys. Res.-Atmos 118, 4001–4024, doi: 10.1002/jgrd.50239 (2013).

