# Supplementary material for: Predicting future uncertainty constraints on global warming projections
Source: Sci Rep. 2016 Jan 11;6:18903. doi: 10.1038/srep18903 (PMC4707548; doi:10.1038/srep18903)
Supplement: Supplementary Information [file srep18903-s1.pdf]

Supplementary information

Predicting future uncertainty constraints on global warming projections

H. Shiogama[1*], D. Stone[2], S. Emori[1], K. Takahashi[3], S. Mori[4], A. Maeda[5], Y. Ishizaki[1] & M. R. Allen[6, 7]

[1]Center for Global Environmental Research, National Institute for Environmental Studies, 16-2 Onogawa, Tsukuba, Ibaraki 305-8506, Japan

[2]Computational Research Division, Lawrence Berkeley National Laboratory, Berkeley, CA 94720, USA

[3]Center for Social and Environmental Systems Research, National Institute for Environmental Studies, 16-2 Onogawa, Tsukuba, Ibaraki 305-8506, Japan

[4]Department of Industrial Administration, Faculty of Science and Technology, Tokyo University of Science, Chiba 278-8510, Japan

[5]Graduate School of Arts and Sciences, The University of Tokyo, 3-8-1 Komaba, Meguro-ku, Tokyo, 153-8902, Japan

[6]School of Geography and the Environment, University of Oxford, OX1 3QY, Oxford, UK

[7]Department of Physics, University of Oxford, OX1 3QY, Oxford, UK

Supplementary Table 1| List of GCMs and the corresponding initial condition ensemble sizes

GCM Name	Initial condition ensemble sizes			
	RCP2.6	RCP4.5	RCP6	RCP8.5
bcc-csm1-1	1	1	1	1
bcc-csm1-1-m	1	1	1	1
CCSM4	6	6	6	6
CSIRO-Mk3-6-0	10	10	10	10
GFDL-CM3	1	1	1	1
GFDL-ESM2G	1	1	1	1
GFDL-ESM2M	1	1	1	1
GISS-E2-H*	3	15	3	3
GISS-E2-R*	3	17	3	3
HadGEM2-ES	4	4	3	3
IPSL-CM5A-LR	4	4	1	4
MIROC5	3	3	3	3
MIROC-ESM	1	1	1	1
MRI-CGCM3	1	1	1	1
NorESM1-M	1	1	1	1

* GISS-ESM2G and GISS-ESM2M include physics perturbation ensemble members as well as initial condition ensemble members.

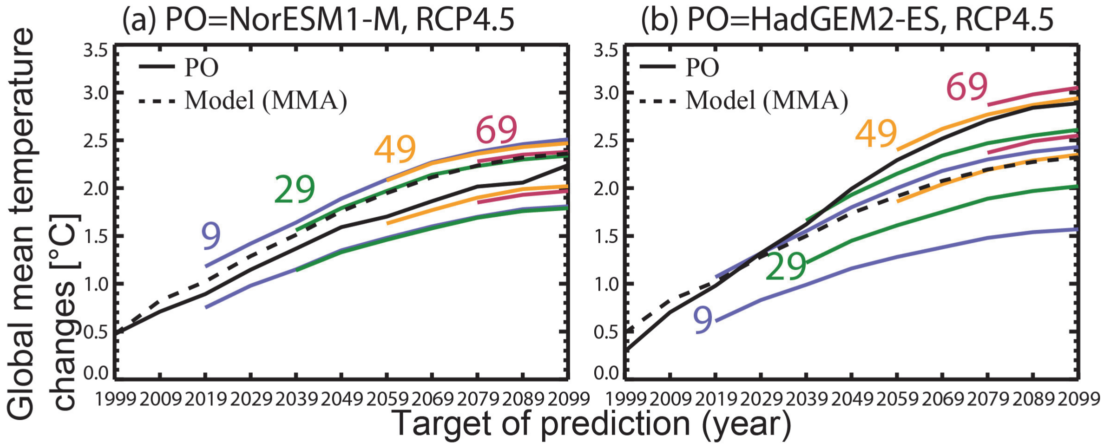

Supplementary Figure 1 | Examples of learning. (a) The black solid line is the Δ*T* of pseudo-observations (POs) from NorESM1-M under the RCP4.5 scenario (°C; relative to the 1900–1919 mean). The black dashed line is the multi-model average (MMA) without the model used for the POs. Colored lines indicate 10–90% ranges of ASK predictions using 80-yr POs up to 2009 (blue), 2029 (green), 2049 (yellow), and 2069 (red). (b) The same as panel (a), but the POs are the HadGEM2-ES runs under the RCP4.5 scenario.

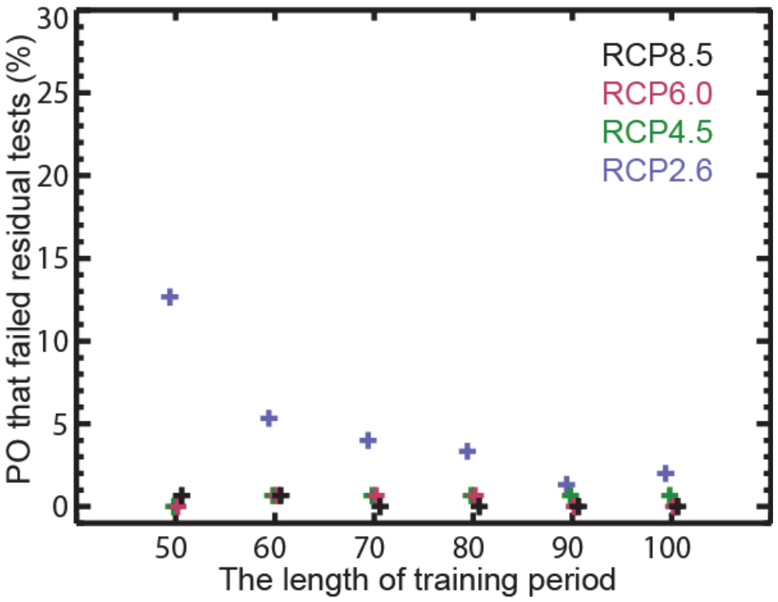

Supplementary Figure 2 | Fractions of POs that the residual variance test failed or for which β was not well constrained for all possible truncation numbers and initial condition ensemble members.

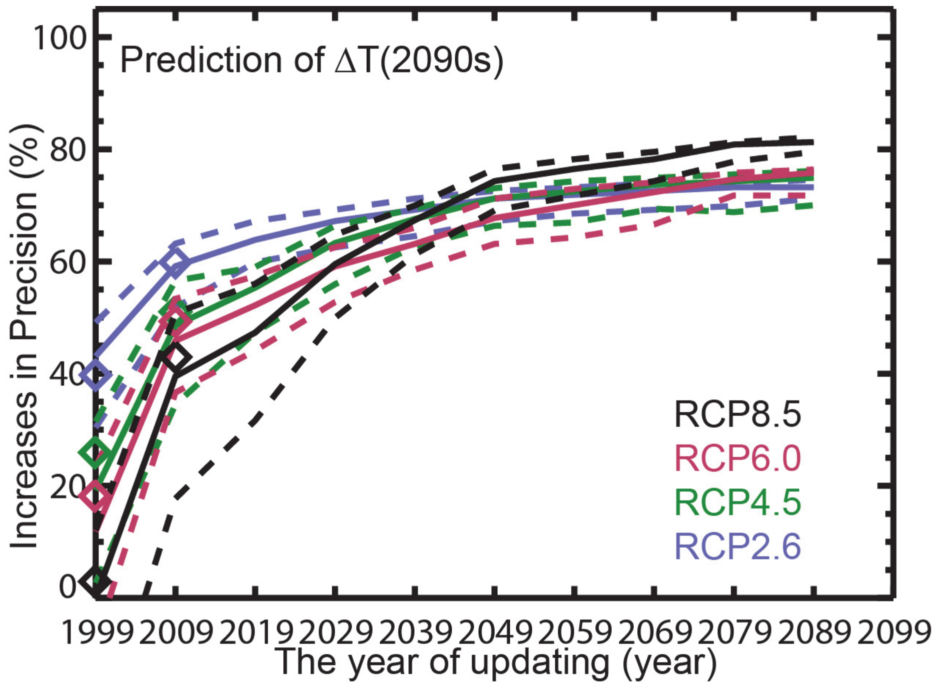

Supplementary Figure 3 | Increases in precision of Δ*T*s in the 2090s. Solid lines indicate increases in precision (%), which are the same as the Fig. 2b. The dashed lines are the 66% uncertainty ranges. Diamonds are the increases in precision using the real observations. We omit the dotted lines indicating the upper limits of precision determined by the internal climate variability.
